# Quantifying gadolinium-based nanoparticle uptake distributions in brain metastases via magnetic resonance imaging

**DOI:** 10.1038/s41598-024-62389-1

**Published:** 2024-05-25

**Authors:** Stephanie Bennett, Camille Verry, Evangelia Kaza, Xin Miao, Sandrine Dufort, Fabien Boux, Yannick Crémillieux, Olivier de Beaumont, Géraldine Le Duc, Ross Berbeco, Atchar Sudhyadhom

**Affiliations:** 1 Brigham and Women’s Hospital|Dana-Farber Cancer Institute|Harvard Medical School, Boston, MA USA; 2 Université Grenoble Alpes, CHU Grenoble Alpes, Service de Radiothérapie, Inserm UA7, Grenoble, France; 3 Siemens Medical Solutions USA Inc., Malvern, PA USA; 4 NH TherAguix SA, Meylan, France; 5 Institut des Sciences Moléculaires, UMR5255, Université de Bordeaux, Bordeaux, France

**Keywords:** Radiotherapy, Nanoparticles

## Abstract

AGuIX, a novel gadolinium-based nanoparticle, has been deployed in a pioneering double-blinded Phase II clinical trial aiming to assess its efficacy in enhancing radiotherapy for tumor treatment. This paper moves towards this goal by analyzing AGuIX uptake patterns in 23 patients. A phantom was designed to establish the relationship between AGuIX concentration and longitudinal (${T}_{1}$) relaxation. A 3T MRI and MP2RAGE sequence were used to generate patient ${T}_{1}$ maps. AGuIX uptake in tumors was determined based on longitudinal relaxivity. AGuIX (or placebo) was administered to 23 patients intravenously at 100 mg/kg 1–5 hours pre-imaging. Each of 129 brain metastases across 23 patients were captured in ${T}_{1}$ maps and examined for AGuIX uptake and distribution. Inferred AGuIX recipients had average tumor uptakes between 0.012 and 0.17 mg/ml, with a mean of 0.055 mg/ml. Suspected placebo recipients appeared to have no appreciable uptake. Tumors presented with varying spatial AGuIX uptake distributions, suspected to be related to differences in accumulation time and patient-specific bioaccumulation factors. This research demonstrates AGuIX's ability to accumulate in brain metastases, with quantifiable uptake via ${T}_{1}$ mapping. Future analyses will extend these methods to complete clinical trial data (~ 134 patients) to evaluate the potential relationship between nanoparticle uptake and possible tumor response following radiotherapy.

**Clinical Trial Registration Number:** NCT04899908.

**Clinical Trial Registration Date:** 25/05/2021.

## Introduction

Modern techniques in radiation therapy (RT) such as intensity modulated radiotherapy (IMRT), volumetric modulated arc therapy (VMAT) and image-guided radiotherapy (IGRT) have revolutionized non-invasive treatments for primary and metastatic solid tumors^1^. Despite significant advances in the field since inception, RT still faces challenges; particularly when targeting tumors near organs at risk (OARs), those difficult to visualize (even with image guidance), or radioresistant tumors^1,2^. The prescribed radiation dose in these cases is often limited to reduce the risk of toxicity to OARs and normal tissue, compromising tumor control probability (TCP) and long-term outcomes^3^. If preferential radio-enhancement could be achieved within tumors, higher doses would not have to come at the cost of normal tissue toxicity and RT could make an important step forward in therapeutic efficacy.

Nanoparticles show promise as both radiation dose and contrast enhancing agents, making them, in effect, theranostic vehicles^4^. Radiation amplification is achieved through photoelectric interactions between the incident clinical radiation therapy and high atomic number (high Z) elements in the nanoparticles^2,5,6^. This effect is greatest for lower photon energies (< 100 keV), a regime that can be augmented in clinical external beam radiation therapy by means of scatter and/or flattening filter free delivery modes. The imaging of nanoparticles can be performed by CT, US, PET, MRI, and optical methods, depending on the nanoparticle composition. The combination of quantitative imaging with in situ radiation dose amplification when beams are applied has the potential to unlock a new era in image-guided radiation therapy.

However, translating theranostic nanoparticles in clinical radiation therapy has been challenging due to issues like consistency, scalable synthesis, and biodistribution^1,7^. Among the very few that have reached the clinic is AGuIX (NH Theraguix, Myelan, France), a gadolinium-based theranostic agent that shows promise in the treatment of previously intransigent tumors^8,9^. The gadolinium provides both radiation dose amplification (Z = 64) and MRI contrast. In vitro studies have demonstrated the radiation amplification effect of AGuIX in multiple cancer cell lines^10,11^. In vivo studies showed preferential uptake of AGuIX in tumors with a corresponding increase in MRI contrast and therapeutic benefit when combined with irradiation^12,13^. Large animal studies demonstrated the safety of AGuIX, even at high doses, and rapid physiological elimination rates in normal tissues comparable to common contrast agents but with double (at minimum) the tumor retention time^10,14^. Unlike traditional contrast agents such as Dotarem or Gadovist, AGuIX exhibits prolonged retention in the tumor. This extended retention is attributed to the enhanced permeation and retention effect (EPR), where nanoparticles access a tumor via its irregular blood vessel structure and remain there due to the tumor's ineffective lymphatic drainage system^15–19^.

The promising preclinical results led to NanoRAD; a Phase 1 clinical trial using AGuIX in humans^8^. This trial assessed the safety and dose tolerance for systemically administered AGuIX in combination with whole brain radiotherapy in patients with multiple brain metastases, who were not suited for stereotactic radiotherapy^8^. Intravenous AGuIX showed no dose-limiting toxicities when administered at doses up to 100 mg/kg and increasing MR signal enhancement was observed in the brain metastases with increasing administered dose^20^. While the primary goal of the trial was to determine safety, a significant correlation was observed between AGuIX uptake in tumors (measured as MRI signal enhancement) and therapeutic response (measured by change in tumor volume)^8^. The success of this Phase 1 trial has led to the opening of several Phase II trials in France and the United States.

In our institution, a double-blinded Phase II clinical trial, NanoBrainMets (NCT04899908), has been opened with the goal of measuring the impact of AGuIX nanoparticles, combined with brain-directed stereotactic radiation, on local tumor control relative to brain-directed stereotactic radiation alone^21^. A critical component in the study of nanoparticle-aided radiation therapy, is to understand the inter- and intra- tumoral relationship between AGuIX uptake and subsequent tumor progression/regression. To this end, we have developed a method for quantification and characterization of AGuIX uptake and patterns thereof using patient MR imaging.

## Methods

### Ethics approval statement

This paper examines data collected from a clinical trial (protocol 20–240, titled "A Double-blind, Phase II Randomized Study of Brain-Directed Stereotactic Radiation With or Without AGuIX Gadolinium-Based Nanoparticles in the Management of Brain Metastases at Higher Risk of Local Recurrence with Radiation Alone") which was approved by the Institutional Review Board (IRB) of the Dana-Farber Cancer Institute. Written informed consent was provided by each individual participant in the trial. This study was conducted in strict compliance with both Dana Farber Cancer Institute and Brigham and Women’s Hospital regulations^21^.

### MRI acquisition

In the NanoRAD phase 1 trial, AGuIX uptake in brain metastases was measured using the Variable Flip Angle (VFA) ${T}_{1}$ mapping method^22^. While the VFA approach has a quick acquisition time, it’s known to be sensitive to B1 inhomogeneities especially at higher field strengths such as 3 T, introducing potential inaccuracies in the ${T}_{1}$ maps^23^. In subsequent work examining NanoRAD data, AGuIX uptake was also quantified using the Magnetization Prepared Rapid Acquisition Gradient Echoes (MPRAGE) sequence^24^. Conventional MPRAGE signals are dependent on ${T}_{1}$ contrast (desired effect), but also on ${M}_{0}$ (proton density) ${T}_{2}^{*}$ and are also prone to bias fields^25^. The MP2RAGE sequence is a variation on the MPRAGE sequence in which two inversion times are acquired during a single acquisition. This results in a robust ${T}_{1}$ mapping technique that cancels out the signal dependency on ${B}_{1}^{-}, {M}_{0}$ and ${T}_{2}^{*}$, leaving an output that is purely ${T}_{1}$ weighted, from which quantitative ${T}_{1}$ values can be estimated^25^.

**Figure 1:**
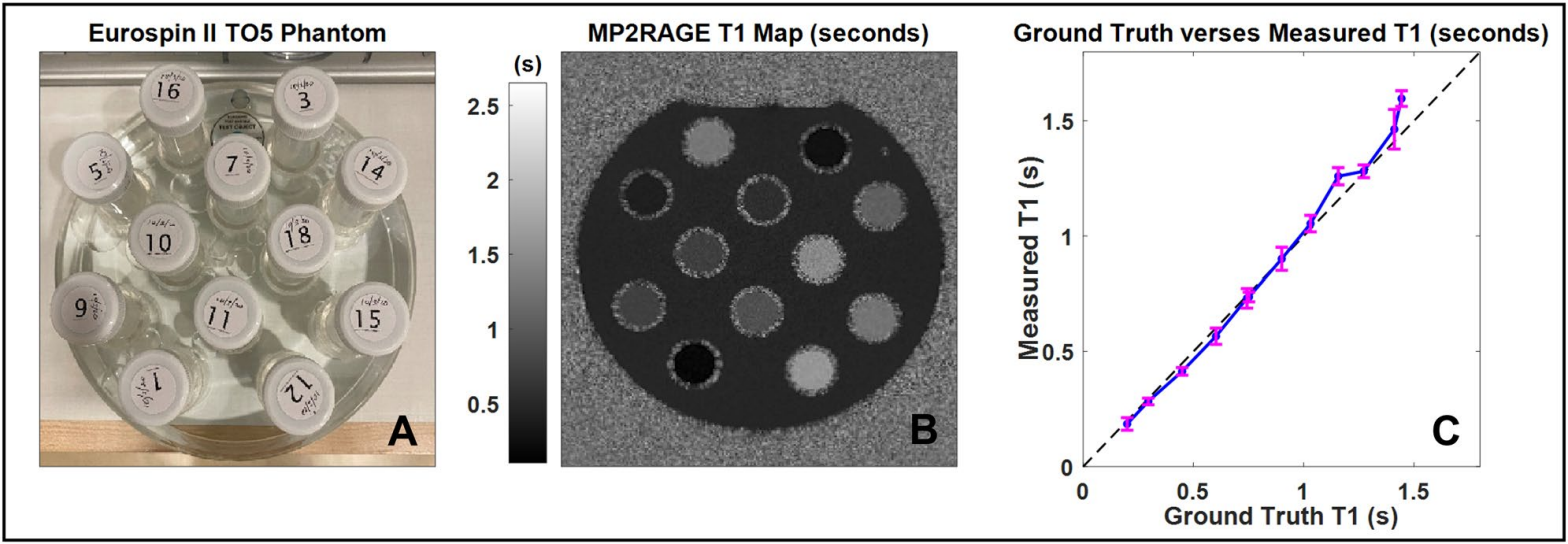
(**A**) The Eurospin II TO5 contrast phantom, which contains 12 vials of unique tissue-mimicking gels of known ${T}_{1}$. (**B**) MP2RAGE generated ${T}_{1}$ map of the Eurospin Phantom. (**C**) Average ${T}_{1}$ values for each vial as generated by MP2RAGE sequence, plotted against ground truth ${T}_{1}$ (values supplied by the manufacturer).

Considering the sensitivities of VFA and the robust nature of MP2RAGE (as compared to MPRAGE), the MP2RAGE sequence was selected for AGuIX quantification throughout the NanoBrainMets clinical trial. Prior to in-human use, the MP2RAGE sequence (parameters detailed in Table 1) was evaluated using the Eurospin II TO5 contrast phantom (Fig. 1). The phantom has 12 cavities in which vials of unique tissue mimicking gels of known ${T}_{1}$ are inserted. The accuracy of each MRI sequence in reflecting the expected ${T}_{1}$ value was tested by extracting average measured ${T}_{1}$ values for each vial and plotting against ground truth ${T}_{1}$ (ground truth values were supplied by the manufacturer). The resulting MP2RAGE ${T}_{1}$ map and accuracy plot can be seen in Fig. 1, where the average error in measured (versus ground truth) ${T}_{1}$ values was 39 ms. These preliminary tests indicated that the MP2RAGE sequence, with parameter values detailed in Table 1, is capable of accurately measuring the quantitative ${T}_{1}$ values of tissue mimicking materials and was therefore utilized in the quantification of AGuIX. A nanoparticle phantom was built and used to determine the relationship between MP2RAGE quantitative ${T}_{1}$ value and AGuIX concentration; this relationship was then used to calculate the uptake in patient scans.

**Table 1:** Relevant MRI parameter values for the MP2RAGE sequence.

MRI parameter	B0 (T)	Time between pulse set (s)	TR (s)	Inversion times (s)	Slice thickness (mm)	Resolution (mm^3^)	No. slices	Flip angles (deg)
GRE 1	3	6.25	0.0071	0.8	1	1	176	4
GRE 2	3	6.25	0.0071	2.2	1	1	176	5

### AGuIX phantom study

A phantom was created to determine the relaxivity of AGuIX, where relaxivity refers to the agent’s ability to enhance relaxation rates compared to an environment without AGuIX^26^. All paramagnetic molecules amplify the nuclear spins' relaxation rate due to the paramagnetic relaxation enhancement (PRE) effect. The relationship between nanoparticle concentration, relaxivity and ${T}_{1}$ change is described for Gadolinium (Gd) in Eq. (1), where ${{T}_{1}}_{AGuIX}$ is of AGuIX-saline solutions, ${{T}_{1}}_{saline}$ is of pure saline, and $r1$ is the longitudinal relaxivity constant. AGuIX concentration is then derived from Gadolinium concentration using Eqs. (2) and (3).

1 $$C(mM Gd)={\frac{1}{r1}}\left({\frac{1}{{T_{1}}_{AGuIX}}}-{\frac{1}{{T_{1}}_{saline}}}\right)$$

2 $$C\left( {\frac{{\text{g}}}{{\text{L}}}~AGuIX} \right) = C\left( {{\text{mM}} ~ Gd} \right)\left( {\frac{{1~ {\text{mol}}~AGuIX}}{{15~{\text{mol}}~Gd}}} \right)\left( {\frac{{10^{{ - 3}} \frac{{{\text{mol}}}}{{\text{L}}}}}{{1~{\text{mM}}}}} \right)\left( {20~{\text{kDa}}} \right)\left( {\frac{{10^{3} \frac{{\text{g}}}{{{\text{mol}}}}}}{{1~ {\text{kDa}}}}} \right)$$

3 $$C\left({\frac{{\text{mg}}}{{\text{ml}}}} AGuIX\right)=C\left({\frac{{\text{g}}}{{\text{L}}}} AGuIX\right)=\left({\frac{4}{3}}\right)\ast C({\text{mM Gd}})$$

**Figure 2:**
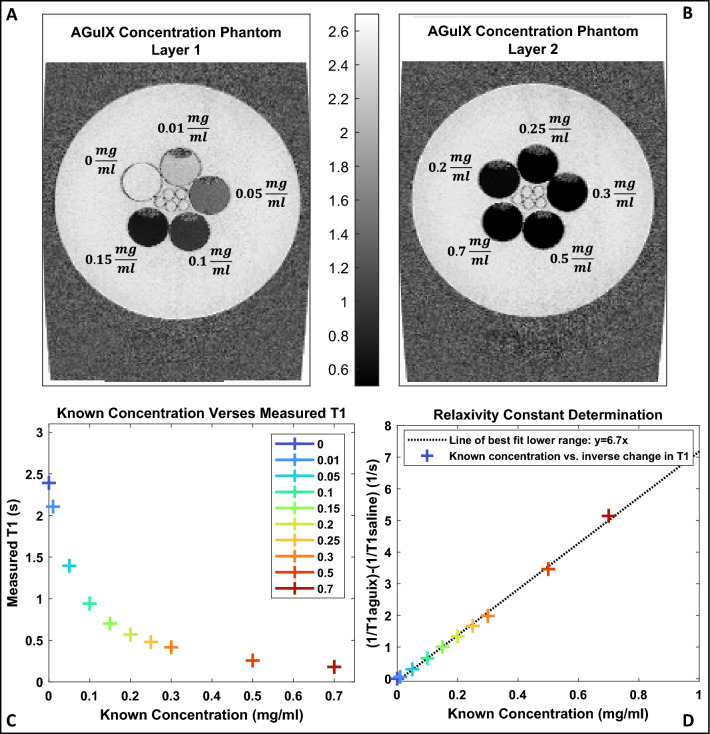
(**A**, **B**) ${T}_{1}$ maps (pixel units are seconds) of the AGuIX concentration phantom consisting of sealed vials of varying AGuIX-saline solutions submersed in water. (**C**) Measured ${T}_{1}$ (average ${T}_{1}$ within each vial containing an AGuIX-saline solution) plotted against known concentration (the concentration each vial was designed to contain). (**D**) Relaxivity plot; inverse change in ${T}_{1}$ plotted against known concentration, where the change in ${T}_{1}$ is calculated using the saline ${T}_{1}$ measurement as baseline and the AGuIX-saline solution ${T}_{1}$ measurements as the change resulting from nanoparticles.

Ten, 25 ml vials were filled with a mixture of saline and AGuIX at varying concentrations (0 mg/ml–0.7 mg/ml) arranged on a vertical axis and placed in a larger container filled with water (detailed in Fig. 2). An MP2RAGE MRI sequence on a 3T MAGNETOM Vida (Siemens Healthcare, Erlangen, Germany) scanner with a 20-channel head/neck coil was used to image the phantom, with the parameters described in Table 1. Respective ${T}_{1}$ maps were calculated in Matlab using qMRLab software, which utilizes a dictionary-based technique whereby ${T}_{1}$ values are extracted from a look-up table that is specific to the scanning parameter values and is a function of B1 and MP2RAGE signal^25,27^. The resulting ${T}_{1}$ maps were used to establish a relaxivity constant (${\varvec{r}}1$) using Eqs. (1)–(3); this process is illustrated in Fig. 2.

### Clinical trial study

Data was gathered from 23 adult patients with brain metastases, each of whom were imaged and treated according to the clinical trial protocol. This paper examines pre-treatment data only; no post-treatment data or outcomes will be reported until after the trial concludes. Specific clinical trial procedures relevant to this paper are detailed as follows.

- 14 days (or less) pre-treatment
  - Planning CT/MRI with standard gadolinium.
- 2–5 days pre-treatment
  - Brain MRI (MP2RAGE sequence) pre-AGuIX infusion.
  - AGuIX infusion (100 mg/kg) or placebo (saline), followed by a 1–5 hour wait time for AGuIX uptake (2 hours preferable).
  - Brain MRI (MP2RAGE sequence) post-AGuIX infusion.
- Treatment Day 1
  - AGuIX infusion (100 mg/kg) or placebo (saline), followed by a 1–5 hour wait time for AGuIX uptake (2 hours preferable).
  - Stereotactic radiosurgery (SRS, 18–20 Gy in 1 fraction) or stereotactic radiotherapy (SRT, 30 Gy in 5 fractions or 25 Gy in 5 fractions).

A 3T MAGNETOM Vida scanner and 20 channel head/neck coil were used to image patients, where the MP2RAGE scanning parameters can be found in Table 1. The clinical images were anonymized and randomly assigned a numeric identifier prior to analysis to maintain the blinded nature of the trial.

**Figure 3:**
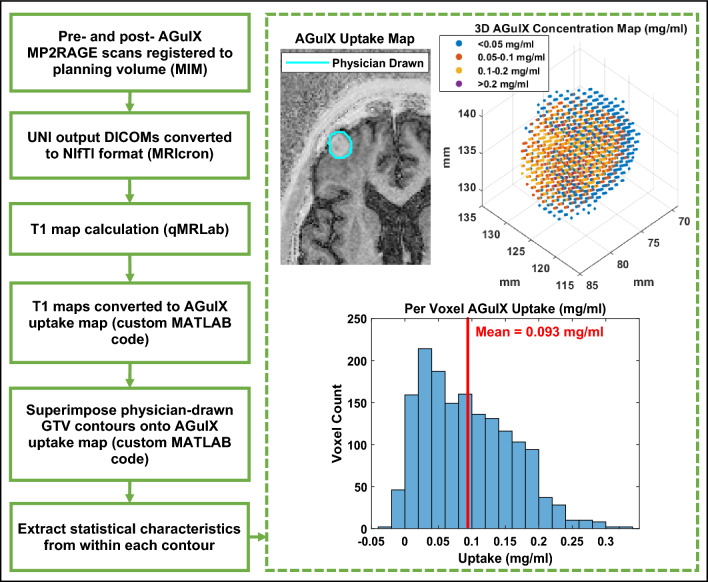
Flow chart illustrating image registration and post processing for each patient and each tumor, where example results are shown within the dashed box.

**Figure 4:**
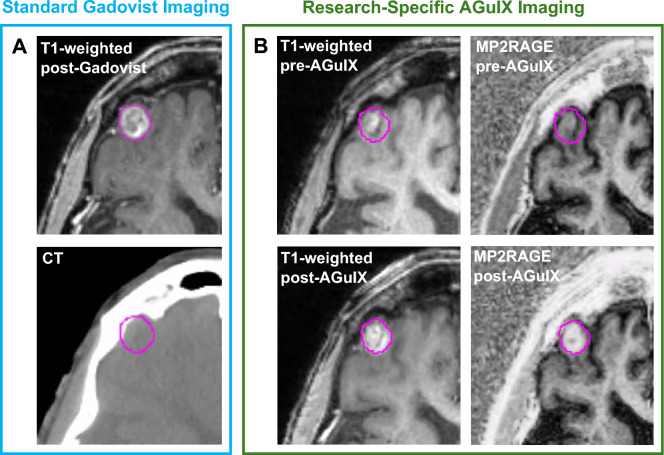
The propagation of tumor delineation from the planning scans to the MP2RAGE output. (**A**) A ${T}_{1}$-weighted scan taken after the administration of Gadovist and the CT scan it was then registered to for treatment planning purposes. (**B**) Both MPRAGE (${T}_{1}$-weighted) and MP2RAGE scans before and after the administration of AGuIX. In each instance, the tumor delineation was propagated from the planning scans to the research specific scans by image registration.

The processing procedure is illustrated in Fig. 3. Physicians used clinical standard post Gd-contrast (i.e. Gadovist) MPRAGE scans from the pre-treatment MRI for target delineation. The propagation of tumor delineation from the planning scans (${T}_{1}$-weighted fused with CT) to the MPRAGE scans (${T}_{1}$-weighted) and finally to the MP2RAGE scans is depicted in Fig. 4. Physicians were blinded to any images obtained with AGuIX. Both the pre- and post- AGuIX MP2RAGE volumes were registered to the planning volume in MIM. Respective pre- and post-AGuIX ${T}_{1}$ maps were calculated using a Matlab-based software, qMRLab^27^. AGuIX concentration maps were derived from ${T}_{1}$ maps using Eqs. (1)–(3), resulting in a per-voxel representation of AGuIX uptake for the entire MRI volume. Tumor-specific uptake (demonstrated in Fig. 3) was examined by applying the physician-delineated GTV contours and considering only the uptake voxels within these boundaries. Uptake within individual tumors was examined through a variety of image processing techniques including morphology and statistical feature extraction.

Due to the double-blind nature of the clinical trial, specific patient-administered agents remain unknown. To differentiate between hypothesized AGuIX and placebo patients, entropy was applied as a classifying image feature. Suspected placebo versus AGuIX patient groups were separated using a k-means clustering algorithm ($k=2$).

## Results

### Phantom study

Figure 2A and B depict MP2RAGE generated ${T}_{1}$ maps of the AGuIX concentration phantom, where the scale is in seconds. As expected, regions in which there are higher concentrations of AGuIX result in shorter ${T}_{1}$ values and therefore present as lower, or darker intensity regions on a ${T}_{1}$ map. The intensity gradient across the lower concentrations (0 mg/ml–0.15 mg/ml) is visually apparent and the intensity gradient across the whole range (0 mg/ml–0.7 mg/ml) is numerically distinguishable.

The relaxivity constant was determined as illustrated in Fig. 2D; Equations 1–3 were rearranged into a $y=mx+b$ format and plotted, where the $y$ term was measured, the $x$ term was known and the slope, or the relaxivity constant, was determined using linear regression. The resulting relaxivity was found to be $r1=6.7 \, \text{mM}^{-1}{\text{s}}^{-1}$. This value is in contrast to relaxivity as calculated in prior AGuIX studies, $r1=8.9 \, \text{mM}^{-1}{\text{s}}^{-1}$, albeit the use of different ${T}_{1}$ mapping sequences (VFA, MPRAGE) and a different batch of AGuIX nanoparticles^8,24,28^.

### Clinical nanoparticle concentration calculation

**Figure 5:**
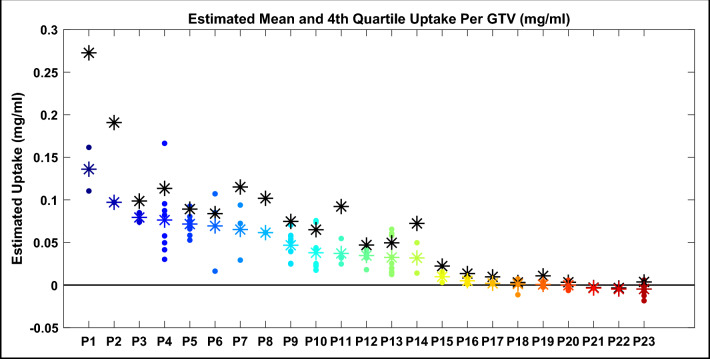
Mean AGuIX uptake for each of 129 tumors in 23 patients (ordered by descending means), where each dot marker represents average AGuIX uptake for individual tumors, each colored star marker represents the average uptake across individual tumors for each patient and each black star marker represents the average upper quartile uptake across individual tumors for each patient.

The clinical trial protocol requires a wait time of 1 to 5 hours between AGuIX/placebo infusion and MRI scan. The maximum time was recorded to be 3 hours and the minimum was 58 minutes, where on average, time for nanoparticle uptake was 92 ± 51 minutes. The mean calculated uptake across 129 cumulative tumors in each of the 23 patients can be seen in Fig. 5, where patients were ordered by descending within-patient average uptake and these average uptake values are represented by colored stars. For patients whose scans are consistent with placebo administration, the average uptake for each patient and all tumors was close to zero (ranging from 0.0097 mg/ml to – 0.0047 mg/ml). This can be observed in Fig. 5, where the placebo patients are predicted to be patients 15 to 23. It can also be observed that some voxels (and therefore some tumor uptake averages) presented with negative uptake values; here we considered negative values to represent an expected amount of noise as well as slight changes in patient positioning and consequent suboptimal registration. For these reasons we considered negative values in all calculations. For patients with contrast enhancement consistent with AGuIX uptake, the average uptake across all patients and all tumors was 0.055 mg/ml, where individual tumor uptake was on average, quite variable both within and between patient tumor groups (average standard deviation of 0.046 mg/ml). The largest individual mean tumor uptake was 0.17 mg/ml (Patient 4) and the smallest mean uptake was 0.012 mg/ml (Patient 13). The average upper quartile uptake for each patient is represented in Fig. 5 by respective black stars; this statistic was calculated to account for a high degree of uptake heterogeneity, even within an individual tumor, as well as to lessen the potential impact of misregistration, which could increase intra-tumor variability. These values, as expected, are generally seen to be higher than the mean uptake values, ranging from 0.047 to 0.27 mg/ml.

**Figure 6:**
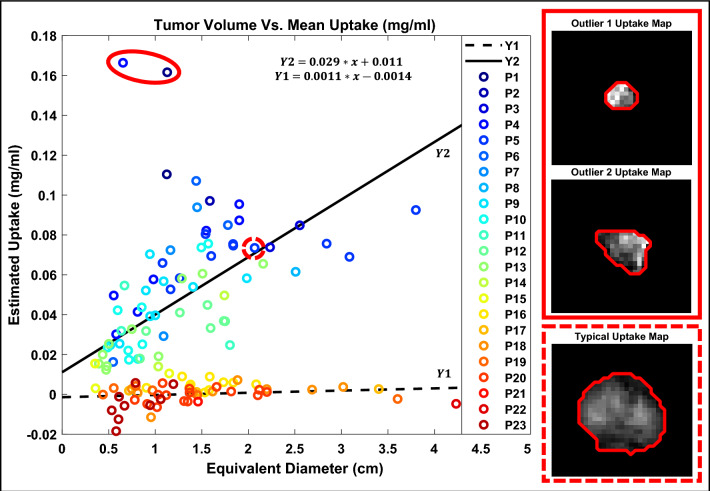
Mean AGuIX uptake for each of 129 tumors in 23 patients plotted against respective tumor sizes, where tumor size is represented by the diameter of a tumor volume equivalent sphere. Examples of tumors with notably high uptake (outliers) can be seen outlined in solid red and an example of a tumor with average uptake can be seen outlined in dashed red. Uptake map cross sections of each example tumor are shown, where uptake is displayed in mg/ml and all are displayed on an intensity scale from 0 to 0.3 mg/ml.

The measured AGuIX concentrations were plotted against tumor size as seen in Fig. 6, where tumor size is represented by the diameter of an equivalent-volume sphere. In patients with uptake consistent with AGuIX administration (as determined by the entropy measure), there appears to be a relationship between tumor diameter and mean concentration. These data were fit linearly, using a robust least absolute residuals technique; the resulting equation had an R-squared value of 0.96 and is illustrated in Fig. 6 (Y2), represented by a solid black line. In contrast, the patients with suspected placebo administration had no relationship between tumor diameter and mean concentration. Exemplar tumor cross sections of respective nanoparticle uptake maps (mapped in units of mg/ml) are also depicted in Fig. 6. Specifically, two tumors that measured as having unusually high uptake (two data outliers) as well as one tumor that could be considered average in the context of the AGuIX uptake data. It can be observed that the two outliers from the suspected AGuIX patients (Y2) were in fact a true representation of unusually high uptake; there is a clear, stark change in intensity with no obvious technical issues causing abnormal measurement. The stronger uptake is clear when compared to the average case in that the change in intensity is visually greater.

**Figure 7:**
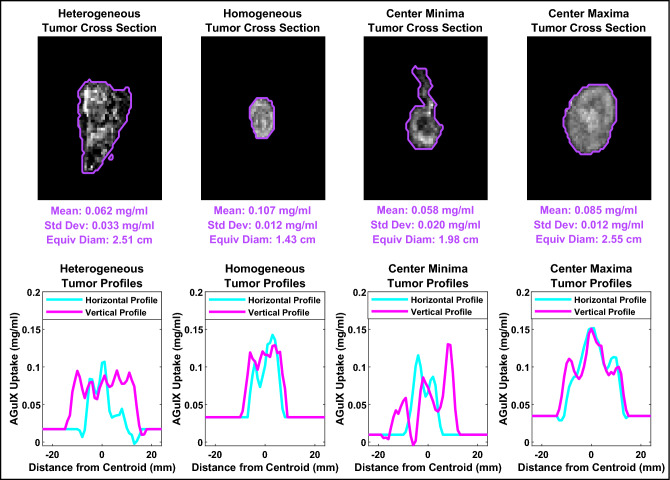
Tumor uptake map cross sections for each type of uptake distribution (heterogeneous, homogeneous, central minima and central maxima) as well as respective horizontal (right–left) and vertical (ant–post) line profiles through tumor centroids. The line profile values outside the tumor boundaries (baseline values) are the averages of normal tissue immediately surrounding the tumors. The mean uptakes, standard deviations and equivalent diameters are provided for each of the tumors displayed.

When examining horizontal and vertical profiles through tumor centers, several different uptake distributions were identified with no consistent pattern across patients. Tumors demonstrated uptake ranging from homogenous to heterogeneous; standard deviations of uptake within tumor volumes ranged from 0.0053 mg/ml to 0.1100 mg/ml. Two patients demonstrated notably lower nanoparticle uptake at the center of each tumor (peak-to-valley ratio of 1.6) and one patient demonstrated notably higher uptake at the center and edges of each tumor (peak-to-valley ratios of 1.8 and 1.2 at the center and edges, respectively). Each of the described patterns can be seen in Fig. 7, along with respective horizontal (right–left) and vertical (anterior–posterior) uptake line profiles.

**Figure 8:**
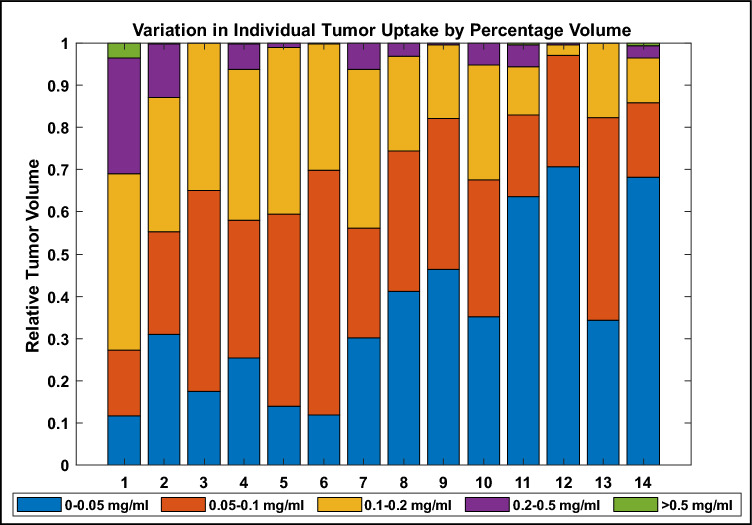
AGuIX uptake distribution within the largest tumor of each patient suspected to have been administered AGuIX (as opposed to placebo), segmented into discrete concentration ranges.

To examine the variation in uptake distributions more thoroughly, the heterogeneity of the tumors was characterized by percent tumor volume at discretized concentrations. Figure 8 depicts this characterization for the largest tumor in each patient suspected to have been administered AGuIX (as opposed to placebo), where the patients are in order of descending average uptake. An overall pattern is visibly apparent; the largest difference between the patients with the highest (Patient 1) and lowest (Patient 14) average uptake is the relative tumor volume at 0.05 mg/ml or less. Where 68% (by volume) of the tumor in Patient 14 is at less than 0.05 mg/ml, only 12% of the tumor in Patient 14 is at the same discretized concentration. Similarly, 73% of Patient 1’s tumor is at a concentration greater than 0.1 mg/ml where only 14% of Patient 14’s tumor has the same level of uptake.

## Discussion

The procedure described herein was used to determine a 3T relaxivity value for AGuIX using MP2RAGE ${T}_{1}$ maps, with a relaxivity of $r1=6.7 \, \text{mM}^{-1}{\text{s}}^{-1}$. This finding differs from the previous, $r1=8.9 \, \text{mM}^{-1}{\text{s}}^{-1}$, possibly due to differences in ${T}_{1}$ mapping sequence accuracies and nanoparticle batches^8^. The MP2RAGE sequence was tested for accuracy in estimating ${T}_{1}$ and results indicated minimal error within the ${T}_{1}$ ranges relevant to this study (Fig. 1). The in-phantom determined relaxivity constant was then used to calculate in-patient tumor uptake and distribution.

Using entropy and k-means clustering, patients were categorized into predicted AGuIX and placebo groups. The predicted AGuIX group presented with markedly higher intra-patient mean uptake and variance than the predicted placebo group, as seen in Fig. 5. Within the predicted AGuIX group, the overall average uptake was determined to be 35% higher than had been found previously (in NanoRAD) for patients receiving 100 mg/kg dose (0.055 mg/ml total average verses a prior 0.036 mg/ml)^8^. This previously reported value was from the NanoRAD dose escalation study for which 3 of 15 patients received a 100 mg/kg dose; a small sample size for which the average uptake is likely to vary with increased patient numbers at the relevant injected dose.

Individual tumor uptake was examined here with respect to size, as illustrated in Fig. 6. The patients predicted to have received placebo all fall at approximately 0 mg/ml regardless of the tumor size. This supports the numerical distinguishability of predicted AGuIX from predicted placebo groups. The predicted AGuIX group appears to have a roughly linear relationship with tumor size at this injected dose (100 mg/kg). Uptake distribution was examined within tumors by means of line profiles; horizontal (left–right) and vertical (ant–post) profiles through each tumor centroid were examined for patterns. These line profiles indicated highly variable intra-tumor accumulation patterns ranging from homogeneous to noise-patterned heterogeneous to localized accumulation (or deficit) at the tumor centers. One of the only prevailing patterns across subjects was that uptake distributions tended to be consistent within each patient; if one tumor demonstrated a lack of uptake at the tumor center then another tumor in the same patient tended to also demonstrate a similar spatial accumulation behaviour. One other prevailing pattern, depicted in Fig. 8, was the increasing percentage of tumor volumes at lower levels of uptake in patients with proportionally lower overall average uptake. The discretized distributions suggest that a higher average uptake might also mean a higher degree of local uptake variability and conversely, a lower average uptake might mean a more homogeneous uptake distribution. Pre-clinical animal studies have indicated AGuIX to have high diffusion potential capable of penetrating and accumulating in an entire tumor volume, including necrotic areas^29^. These studies also indicate a progression of distribution patterns as AGuIX accumulates in a lesion or an organ over time^10^. Differences in accumulation times (from AGuIX infusion to MRI scan) may mean patients are imaged at different spatial distribution progression stages. Future studies with serial imaging in the minutes after infusion may help to elucidate the time dynamics of how AGuIX diffuses into tumors. Other factors at play may include differences in tumor characteristics (e.g., vascularization or interstitial pressure) and patient physiologies (blood flow, metabolism, overall health), amongst other variables.

The GTV delineations were subject to some degree of uncertainty due to the transfer of contours (via registration) across images and modalities; this is particularly true for smaller tumors for which misregistration uncertainty has a larger impact on voxel-based uptake estimation. In addition, GTV volumes were determined using a standard Gd-based contrast agent (i.e. Gadovist) MRI a few days before AGuIX administration, whereas AGuIX uptake may have different spatial accumulation patterns. As a result, tumor surfaces, as delineated by the GTV and as reflected in data presented here may present larger than actual ${T}_{1}$ variances. An example of tumor contour propagation from the planning scans to the MP2RAGE scans can be seen in Fig. 4, for which the regions of uncertainty are at the contour edges. Measurements derived in this paper aimed to mitigate the described variation by considering averages. Auto-contouring may be a useful tool in future analysis for adapting physician delineated GTVs to account for possible errors in misregistration, patient positioning or movement, anatomy changes and even differences in bioaccumulation patterns between standard contrast agents and AGuIX.

## Conclusion

We have established a robust method to quantify AGuIX uptake in brain metastases (on a per voxel basis) using MP2RAGE generated ${T}_{1}$ maps. Quantification has important clinical implications, enabling more precise targeting and in situ radiation dose amplification. The results of this study pave the way for a transformative approach in radiation therapy where nanoparticle distributions inform and optimize treatment planning, embodying a truly theranostic paradigm.

## Data Availability

The datasets used and/or analyzed during the current study are available from the corresponding author on reasonable request.
