# Author Correction: Quantifying gadolinium-based nanoparticle uptake distributions in brain metastases via magnetic resonance imaging

**DOI:** 10.1038/s41598-025-90982-5

**Published:** 2025-03-04

**Authors:** Stephanie Bennett, Camille Verry, Evangelia Kaza, Xin Miao, Sandrine Dufort, Fabien Boux, Yannick Crémillieux, Olivier de Beaumont, Géraldine Le Duc, Ross Berbeco, Atchar Sudhyadhom

**Affiliations:** 1 Brigham and Women’s Hospital|Dana-Farber Cancer Institute|Harvard Medical School, Boston, MA USA; 2 Université Grenoble Alpes, CHU Grenoble Alpes, Service de Radiothérapie, Inserm UA7, Grenoble, France; 3 Siemens Medical Solutions USA Inc., Malvern, PA USA; 4 NH TherAguix SA, Meylan, France; 5 Institut des Sciences Moléculaires, UMR5255, Université de Bordeaux, Bordeaux, France

Correction to: *Scientific Reports* 10.1038/s41598-024-62389-1, published online 25 May 2024

In the original version of this Article, the authors omitted the below Reference, which is now as Reference 9.

9. Maury, P., Mondini, M, Chargari, C., Darricau, A., Shahin, M., Ammari, S., Bockel, S., Genestie, C., Wu, T.-D., Lux, F., Tillement, O., Lacombe, S., Deutsch, E., Robert, C. & Porcel, E. Clinical transfer of AGuIX®-based radiation treatments for locally advanced cervical cancer: MR quantification and in vitro insights in the NANOCOL clinical trial framework. *Nanomed. Nanotechnol. Biol. Med.* **50**, 102676, ISSN 1549-9634. 10.1016/j.nano.2023.102676 (2023).

As a result, in the Introduction section,

“Among the very few that have reached the clinic is AGuIX (NH Theraguix, Myelan, France), a gadolinium-based theranostic agent that shows promise in the treatment of previously intransigent tumors^8^.”

now reads:

“Among the very few that have reached the clinic is AGuIX (NH Theraguix, Myelan, France), a gadolinium-based theranostic agent that shows promise in the treatment of previously intransigent tumors^8,9^.”

As a result of the changes, the References have been renumbered.

The original Article has been corrected.

